# Bromodomain inhibitors a decade later: a promise unfulfilled?

**DOI:** 10.1038/s41416-020-01079-x

**Published:** 2020-09-29

**Authors:** Monica M. Mita, Alain C. Mita

**Affiliations:** The Samuel Oschin Comprehensive Cancer Institute, Cedars-Sinai Medical Center, Los Angeles, CA USA

**Keywords:** Drug development

## Abstract

Over the last decade, bromodomain inhibitors have emerged as a promising class of anticancer drugs. However, the clinical progress of these agents has faced significant obstacles, which precluded their regulatory approval. This editorial will review the challenges and opportunities associated with the development of bromodomain inhibitors.

## Main

One of the most studied hallmarks of cancer is epigenetic modulation, which has been shown to induce tumorigenesis as well as resistance to standard-of-care treatments.^1^ Considerable research efforts were focused on bromodomains (BRDs), a family of evolutionarily conserved motifs identified in the early 1990s in the *Brahma* gene of *Drosophila melanogaster*.^2^ BRDs bind acetylated lysines in histone tails and regulate gene transcription by the recruitment of molecular partners. They are commonly subclassified into bromodomain and extra-terminal (BET) and non-BET families. Based on their biology and deregulation in different tumour types, BRDs appeared as a promising target for anticancer drug development, and several BRD inhibitors entered clinical trials over the last decade.

In this issue, Ameratunga et al.^3^ present a well-executed Phase 1 study with ODM-207, a novel pan-BET inhibitor with a distinct chemical structure compared with the first-generation BET inhibitors.^4^ The study enrolled a relatively large number of patients, and the investigators used a novel study design allowing almost all patients to be treated at or close to the recommended dose. Dose-limiting toxicities (DLTs) were mainly cumulative fatigue and nausea with other adverse events including gastrointestinal (GI) symptoms, anorexia and thrombocytopenia. It is noteworthy that some of the DLTs occurred outside of the 28-day DLT window. The drug’s pharmacokinetic (PK) behaviour is dose-dependent, but the therapeutic window is narrow. Interestingly, thrombocytopenia, a well-known side effect of BRD inhibitors, was used as a pharmacodynamic (PD) biomarker. This is because it occurred relatively rapidly after dosing and typically resolved shortly and rapidly after treatment interruption. Even though most patients received pharmacologically active doses, no partial or complete responses were seen, and around 30% of patients had stable disease. The perspectives for further development of OMD207 include different combinations; however, more preclinical work is probably needed to identify tumour subtypes more sensitive to the drug. The lack of a clear path for regulatory approval has been a common thread for other similar drugs.

A number of studies with different small molecules inhibiting bromodomains (either BET or non-BET) have been performed over the last few years, and several reviews have described the structures, mechanism of action and preliminary preclinical and clinical data associated with these drugs.^5–7^ Although their chemical structure is diverse, common trends are now emerging from these studies. BRD inhibitors exhibited similar toxicity profiles, with thrombocytopenia, fatigue and GI toxicity (mainly diarrhoea) emerging as “class effects.” They have also shown less-than-anticipated efficacy; their antitumour effect was observed mainly in haematological malignancies and rare diseases such as NUT, and multiple mechanisms of resistance have already emerged. Disappointingly, none of the BRD inhibitors has yet received regulatory approval, which brings several questions regarding their development and potential as anticancer therapies. First, BRDs are a structurally and functionally complex class, with additional layers of subtle regulations based on various isoforms of each BRD protein (produced by alternative splicing) and differences of expression and functionality in diverse tumour types. Second, and not surprising, it is now clear that BRDs inhibitors are not the “magic bullet”, cure-all-cancers as an overoptimistic initial evaluation may have perceived them. They are rather closer in their mechanism of action to targeted therapies, and likely to work only in selective cancer subtypes. Hence, it is important to further understand the biology of the driver oncogenes regulated by BRDs in distinct tumour cells, such as *c-MYC*, *NF-kb*-dependent genes, *BCL* family etc. This will potentially allow to screen patients for enrichment in clinical trials. Third, the importance of the chemical structure and underlying nuances of BRD inhibition are unclear at this time, and specifically the superiority of either class (BET vs non-BET or selective BET vs pan-BET inhibitors) remains to be demonstrated.

Given the relative lack of success and the exposed underlying missteps in the often-too-hasty preclinical and clinical development of BRD inhibitors, a course change appears imperative. More in-depth preclinical and translational research seems a logical first step in order to better understand the specific tumours where BRD inhibitors are more likely to be successful. Having access to patient-derived tumour tissue and studying super-enhancer activity before and after treatment, analysing downstream protein translation after use of BRD inhibitors, studying in parallel other pathways that could play a role in either enhancing or reducing the antitumour effect and identifying novel PD biomarkers represent important and much needed strides to better understand the use, efficacy as well as combinatorial potential of this class of agents. In-depth analysis of the PK profile and its correlation with both toxicity (as presented in the Ameratunga manuscript^3^) and efficacy is also of significant importance since chronic administration is based on acceptable toxicity and expected PK/PD activity with the chosen dose. Concerted translational work in early clinical trials (and ideally in Phase 1 trials) for elucidating the molecular mechanisms of tumour response will allow enrichment in Phase 2–3 studies and could provide an efficacy signal that could be lost in trials performed in unselected populations. Lastly, translational work will also help to identify potential rational combination partners or solutions to prevent resistance. Recent publications from preclinical work reveal a potential for combination with immunotherapies, PARP and CDK inhibitors, opening the door to possible studies in selected patient populations.^8–11^

In conclusion, there is no doubt that BRD inhibitors have an untapped potential as anticancer drugs. However, there is no obvious “low-hanging fruit” for their development, which is clearly more complex than anticipated. As we are looking ahead at the next step, the question is do we have a leading candidate for BRD inhibitors? After evaluating the science and clinical evidence, the ideal candidate will be the one with a strong preclinical background, mechanistic proof-of-principle established by tumour biopsies and clinical evidence in Phase 1, optimised dosing schedule allowing chronic use and finally, good potential for rational combinations. We confidently look forward to the research community to meet these expectations.

## Data Availability

Not applicable.
